# Supplementary material for: Study of the Effect of Wild-Type and Transiently Expressing CXCR4 and IL-10 Mesenchymal Stromal Cells in a Mouse Model of Peritonitis
Source: Int J Mol Sci. 2023 Dec 30;25(1):520. doi: 10.3390/ijms25010520 (PMC10778615; doi:10.3390/ijms25010520)
Supplement: Supplementary file 1 [file ijms-25-00520-s001.zip › ijms-2759124-Supplementary Tables.pdf]

Supplementary Table S1

ANIMAL WELFARE SUPERVISION PARAMETERS
**1.- WEIGHT LOSS (DAILY)**<br>1) Normal weight<br>2) < 10%<br>3) Between 10 -19%<br>4) > 20%
**2.-PHYSICAL APPEARANCE OF THE ANIMAL**<br>1) Normal<br>2) Bristly, dull and/or dirty coat.<br>3) One or more of the following signs: mucous and/or bloody discharge from any opening, diarrhea, detectable enlarged organs (lymph nodes, spleen, liver)<br>4) One or more of the following signs: abdominal distension of any origin, presence of ascites involving an increase of more than 10% of the initial body weight, dyspnea (particularly if accompanied by nasal discharge and/or cyanosis), cachexia
**3.- BEHAVIORAL DISORDERS**<br>1) None<br>2) Difficulty getting around normally<br>3) Difficulty in reaching food/drink. Isolation from the rest of the animals in the cage.<br>4) Decreased movementl ntention to "hide" in the shavings, does not respond to stimuli, lethargy.<br>7) Comatose state
**4.- DEHYDRATION**<br>1) None<br>2) Mild<br>3) Severe
**5.- SEVERE ANAL SPHINCTER IRRITATION**<br>1) No<br>3) Yes

Supplementary Table S2

HISTOPATHOLOGICAL EVALUATION OF THE INTESTINAL LOOPS						
MEANS±SD OF THE INFLAMMATORY CELLS IN THE CECUM (POINT OF THE CLP)*						
	CG		*hASC*		*hASC-MOD*	
	5 days	***11 days***	***5 days***	***11 days***	***5 days***	***11 days***
Neutrophils	26.4±21.6	19.1±6.9	13.5±6.4	11.7±4.1	30.4±17.1	10.8±6.4
Plasm cells	5.8±2.7	10.8±6.1	4.7±2.9	10.7±1.9	13.4±5.9	28.1±6.1
Total number Macrophage (CD68+)	20.05±12.5	18.1±9.7	22.05±7.9	14.4±4.5	6.6±3.13	13.1±8.1
Macrophage M2 (CD163+)	0.05±0.6	5.65±4.7	7.2±3.2	7.85±5.1	2.6±2.9	6.1±4.6
						**P <0.05.*
**Tissue Damage in the Cecum Layers** *(Grading changes: 0 = none, 1 = minimal, 2 = mild, 3 = moderate, 4 = severe)*						
Serosal Layer	4	4	2	2	3	2
External Muscle Layer	4	4	2	1	2	1
Internal Muscle Layer	4	4	2	1	2	1
Mucosal Layer	4	4	2	1	2	1
Tissue Damage in the Colon and Small Intestine Layers *(Grading changes: 0 = none, 1 = minimal, 2 = mild, 3 = moderate, 4 = severe)*						
Serosal Layer	4	4	2	2	3	2
External Muscle Layer	2	2	0	0	2	0
Internal Muscle Layer	0	0	0	0	0	0
Mucosal Layer	0	0	0	0	0	0

Supplementary Table S3

HISTOPATHOLOGICAL EVALUATION OF THE SPLEEN						
	Control Group		ASCs		ASCs-MOD	
	5 days	11 days	5 days	11 days	5 days	11 days
**PALS**						
Variation of size	↑4	↑4	↑2	↑1	↑2	↑2
Variation of number of cells	↑4	↑4	↑2	↑1	↑2	↑2
Variation of number lymphocytes	↑4	↑4	↑2	↑1	↑2	↑2
**MARGINAL ZONE**						
Variation of size	↑3	↑3	↑1	↑2	↑1	↑1
Variation of number lymphocytes	↑3	↑3	↑1	↑2	↑1	↑1
**FOLLICLES**						
Variation of number	↑2	↑2	↑1	↑2	↑1	↑2
Variation of number lymphocytes	↑2	↑2	↑1	↑2	↑1	↑1
Increased/decr. of Germinal Centers	↑1	↑1	0	↑2	↑2	↑1
**MACROPHAGES**						
Total Macrophages (CD68+) (*MEAN CEL/400x FIELD*)*	51.7±17.2	51.2±16.9	24.6±8.3	30.8±9.5	49.4±13.2	51.05±16.4
M2 Macrophages (CD163+).(MEAN CEL/400x FIELD)*	10.8±6	11.8±6.5	9.55±4.7	13.65±5.8	20.1±11.1	23.75±7.9
						**P* <0.05.
**FIBROSIS**	0	0	0	0	0	0
**NECROSIS**	0	0	0	0	0	0
